# Energy Bandgap and Edge States in an Epitaxially Grown Graphene/*h*-BN Heterostructure

**DOI:** 10.1038/srep31160

**Published:** 2016-08-09

**Authors:** Beomyong Hwang, Jeongwoon Hwang, Jong Keon Yoon, Sungjun Lim, Sungmin Kim, Minjun Lee, Jeong Hoon Kwon, Hongwoo Baek, Dongchul Sung, Gunn Kim, Suklyun Hong, Jisoon Ihm, Joseph A. Stroscio, Young Kuk

**Affiliations:** 1 Department of Physics, Seoul National University, Seoul 151-747, Korea; 2 Department of Physics, Sejong University, Seoul 143-747, Korea; 3 Center for Nanoscale Science and Technology, National Institute of Standards Technology, Gaithersburg, MD 20899, USA

## Abstract

Securing a semiconducting bandgap is essential for applying graphene layers in switching devices. Theoretical studies have suggested a created bulk bandgap in a graphene layer by introducing an asymmetry between the A and B sub-lattice sites. A recent transport measurement demonstrated the presence of a bandgap in a graphene layer where the asymmetry was introduced by placing a graphene layer on a hexagonal boron nitride (*h*-BN) substrate. Similar bandgap has been observed in graphene layers on metal substrates by local probe measurements; however, this phenomenon has not been observed in graphene layers on a near-insulating substrate. Here, we present bulk bandgap-like features in a graphene layer epitaxially grown on an *h*-BN substrate using scanning tunneling spectroscopy. We observed edge states at zigzag edges, edge resonances at armchair edges, and bandgap-like features in the bulk.

Since its first successful isolation, graphene has been considered as a material suitable for future application in signal-switching devices with spin-sensitive transport^1^. However, the on-off ratio of the fabricated switching devices is rather low because of the semimetallic nature of graphene, which is caused by the massless Dirac-fermion dispersion^2^. It was suggested that a bandgap can be produced if two different local potentials are applied to the A and B sites of graphene in a honeycomb lattice^1^,^3^,^4^,^5^. The electronic structure that results from this sub-lattice asymmetry can be explained in terms of a Dirac-fermion dispersion with a mass term^1^,^5^. This sub-lattice asymmetry is present in a graphene bilayer^3^. A bilayer device can be useful because the gap is tunable when an electric field is independently applied to the top and bottom layers. Moreover, a similar device can be produced by replacing the bottom layer with hexagonal boron nitride (*h*-BN)^4^,^6^,^7^. This can be achieved by mechanical placement or epitaxial growth of a graphene layer on an *h*-BN substrate. In a recent transport study, the presence of a minigap at the neutral point in a graphene layer on an *h*-BN substrate was confirmed^5^.

In a graphene layer mechanically placed on *h*-BN, the valley degree of freedom was found to remain intact^5^,^8^,^9^,^10^; therefore, most electronic and transport characteristics of a free-standing graphene layer are preserved. For example, sharp edge states should be present at the Dirac point in a graphene nanoribbon (GNR) with zigzag-edge terminations^11^,^12^. The peak height of these edge states decreases with increase in GNR width; however, the peak is still noticeable in the graphene nano islands (GNIs) with one relatively long zigzag edge^11^. When the spin degree of freedom is considered in^13^,^14^,^15^,^16^, a bandgap locally develops at the edge because either a ferromagnetic or an antiferromagnetic state becomes the ground state. This edge state with the band gap may decay spatially away from the edge with a finite decay length if there is no corresponding bulk state^17^. However, if there is a corresponding bulk state, this edge state may merge with the bulk state. This is called an edge resonance instead of edge state, and it is analogous to a surface resonance.

Scanning tunneling microscopy (STM) is an ideal tool for both imaging local geometric structures and observing local electronic structures. While the above theoretical predictions were derived for a free-standing graphene layer, most previous STM studies were performed with graphene flakes epitaxially grown or drop-cast on metal substrates. Energy gaps of 0.2–0.3 eV were reported in a GNR that was drop-cast on Au(111)^18^, a graphene monolayer (ML) epitaxially grown on Cu/Ir(111)^19^, and a graphene bilayer epitaxially grown on Ru(0001)^20^. It has been theoretically established that the gap opening in a graphene ML on a metal substrate can also be explained by the hybridization of the metal substrate bands to the graphene valence band^19^. Tao *et al*.^21^ reported evidence of edge states at a chiral edge 16.1° off from the zigzag direction in GNRs that were drop-cast on an Au(111) surface. Using scanning tunneling spectroscopy (STS), they observed double peaks at the GNR edges near the Fermi level and attributed their presence to the zigzag edge states. Other theoretical and experimental studies^22^,^23^,^24^ have demonstrated that unsaturated σ orbitals at the graphene edge bond to metal substrate atoms and that the edge state predicted in free-standing graphene may be substantially reduced or absent in graphene on metal substrates. Our results for a graphene layer on *h*-BN may better mimic those for a freestanding graphene layer than those for a graphene layer on a metal surface. One may notice that most theoretical predictions were done for a freestanding graphene layer.

In this study, we performed atomically resolved electronic structure measurements in epitaxially grown GNIs on an epitaxially grown *h*-BN ML on a Cu(111) surface. Edge states of the graphene layer and bulk gap-like features were observed in this pure and well-defined *heterostructure.*

## Results

### Growth of Graphene on *h*-BN

**Figure 1:**
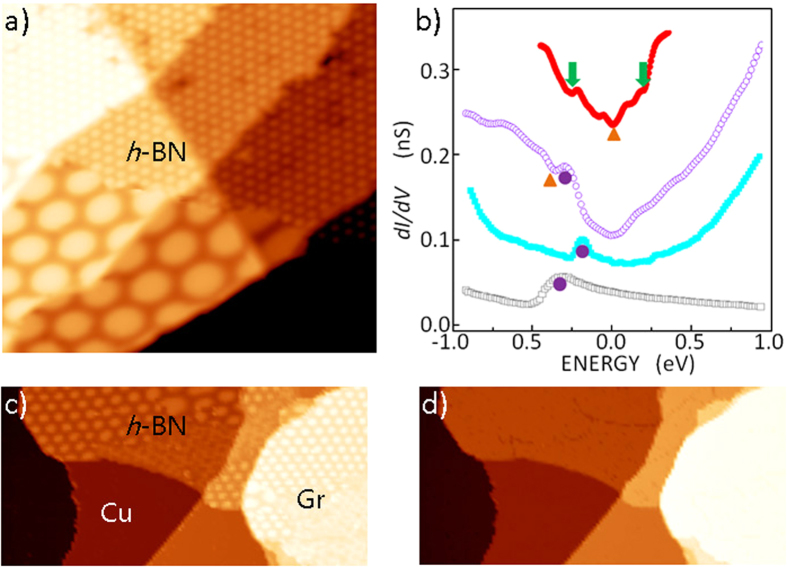
Epitaxial growth of the *h*-BN and graphene layers and their identification (**a**) STM topography of a (70 × 70) nm^2^ *h*-BN covered surface obtained with *V*_*s*_ = 1.0 V, and *I*_*t*_ = 0.15 nA. (**b**) Scanning tunneling spectra of a clean copper substrate (gray open squares), *h*-BN grown on Cu(111) (full light-blue squares), graphene grown on Cu(111) (open purple circles), and graphene grown on *h*-BN/Cu(111) (full red circle). The purple dots represent copper surface peaks, the brown triangles denote Dirac points, and the green arrows indicate moiré potential-induced peaks (**c,d**) STM topography of a graphene layer grown on an (52 × 26) nm^2^ *h*-BN surface epitaxially grown on a Cu(111) surface in area obtained with *V*_*s*_ = 4.0 V (**c**), 0.7 V (**d**) and *I*_*t*_ = 0.15 nA. Moiré patterns in the *h*-BN layer are observed at the bias of 4.0 V.

**Figure 2:**
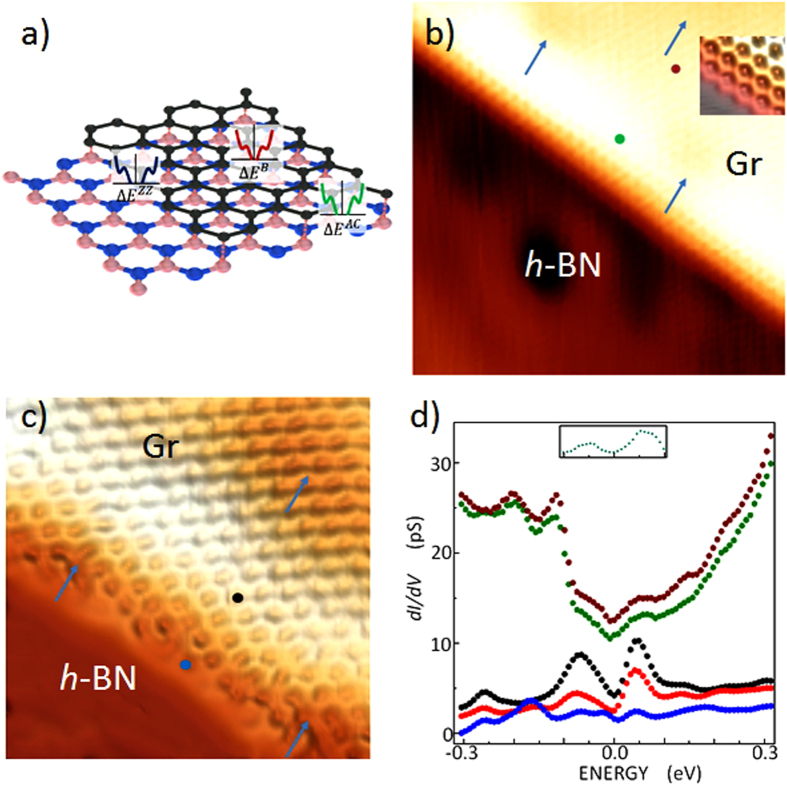
STM topography and STS near zigzag and armchair edges. (**a**) Three different energy bandgaps in a graphene layer epitaxially grown on *h*-BN/Cu(111) are illustrated as bulk bandgap, zigzag edge gaps, and armchair edge resonance. B stands for the bulk, ZZ for zigzag, and AC for armchair. (**b**) A (6.1 × 6.1) nm^2^ STM topography near a zigzag edge obtained with *V*_*s*_ = 0.1 V, and *I*_*t*_ = 15 nA. In the magnified inset image, bright spots at the center of the honeycomb lattice are visible in the 3D representation of a close-up view of this zigzag edge. Blue arrows indicate hollow sites in moiré patterns. (**c**) STM topography near an armchair edge of a (3.5 × 3.5) nm^2^ graphene layer obtained with *V*_*s*_ = 0.01V, and *I*_*t*_ = 15 nA. (**d**) Scanning tunneling spectra at five lattice constants away from a zigzag edge (full green circles), 2.4 nm away from the zigzag edge (full dark-red circles). Those at an armchair edge (full blue circles), five lattice constants away from the armchair edge (full black circles), five lattice constants away from another armchair edge (full red circles: the location is not shown in Fig. 2c). An inset spectrum indicates a spectrum after subtracting a linear Dirac dispersion and a parabolic tunneling background in a tip-sample geometry. The estimated errors of energy are ± 0.02 eV.

We produced a graphene layer on an *h*-BN ML grown on a Cu(111) substrate. After growing approximately half of the *h*-BN ML on a Cu(111) surface using borazine (B_3_H_6_N_3_), the incoming graphene precursor gas (ethylene) was decomposed on the exposed half of the Cu(111) surface. Most graphene layers were grown on the exposed Cu substrate, while some were grown as GNIs on the *h*-BN layer. Figure 1a shows an area covered with the epitaxially grown *h*-BN layer, which showed moiré patterns with many different periods, similar to earlier results^25^. The identity of the grown layer was confirmed by the STS results, as shown in Fig. 1b. The growth rate of graphene on an *h*-BN surface is ≈1/1000 times than that achieved on a Cu(111) surface. A large dosage (>10^8^ L) of precursor gas was used to grow graphene layers on *h*-BN^26^,^27^; the grown graphene layers were flat but contained many impurity atoms. In our study, upon exposure to 10^3^–10^4^ L of ethylene, small GNIs were nucleated on the *h*-BN surface. The produced GNIs on the *h*-BN surface were scarce, but they were pure and contained no traces of impurities. The GNIs grown on *h*-BN were usually located near the *h*-BN or Cu step edges, as shown in Fig. 1c,d, S1, and S2; the edges may serve as supply channels for the diffusion of carbon atoms that decompose on the exposed Cu substrate. We believe that all graphene edges were terminated by hydrogen atoms generated in the process of ethylene decomposition. The STM topography of a graphene layer on *h*-BN is usually featureless at a sample bias of −3–3 eV. Figure 1d shows a GNI on *h*-BN at a sample bias of 0.7 V, revealing a featureless flat island. As the bias was increased to 4.0 V, moiré patterns under the top-layer graphene became visible, as shown in Fig. 1c. Atomic details and moiré patterns were visible when the surface was imaged at a bias below 0.1 eV (Fig. 2b,c).

The identity of the grown layers was confirmed by the STS spectra. The spectra in Fig. 1b are representative; their major peaks were well-reproduced, but detailed curvatures varied with different tips and the gaps between a probing tip and a sample. The Cu substrate was clearly identified by its surface state peaks, which are displayed as purple dots in Fig. 1b. As reported earlier^28^,^29^,^30^, the spectrum of graphene/Cu(111) differs from that of *h*-BN/Cu(111), as demonstrated by the different shifts of surface peaks due to different charge transfer from Cu substrate to overlayers: ≈−0.3 eV for graphene, and ≈−0.2 eV for *h*-BN. A weak dip at ≈−0.4 eV originated from the Dirac point in the graphene layer on Cu(111). In addition to the shifted Cu surface states, the *h*-BN spectrum had no distinctive features other than ≈4 eV bandgap of the *h*-BN layer (not shown in Fig. 1b). The STS spectrum of the graphene layer on *h*-BN was rather different from that on Cu(111), as shown in Fig. 1b and as previously reported^8^,^28^. The Dirac point was located at the Fermi level. Two peaks located at ≈−0.25 eV and ≈0.2 eV were produced by the long-range moiré potential in a graphene layer with a moiré period of ≈3 nm^8^.

### Bandgap

**Figure 3:**
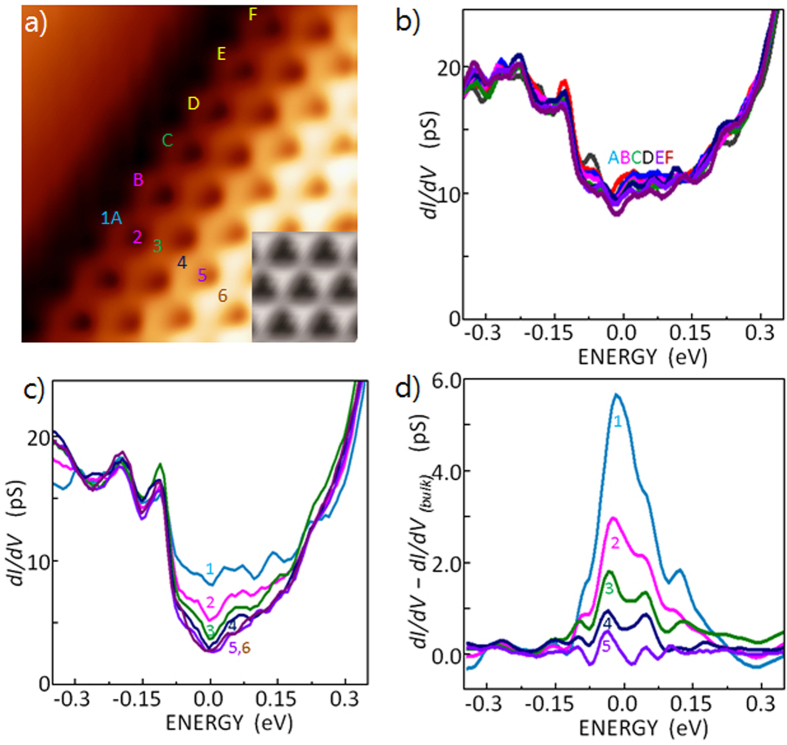
STM topography and STS of graphene layer on *h*-BN grown on Cu(111) (**a**) A (1.6 × 1.6) nm^2^ STM topography of a graphene layer on *h*-BN obtained with *V*_*s*_ = 0.01 V, and *I*_*t*_ = 15 nA. A simulated image obtained with an *ab initio* calculation is shown in the bottom right corner. (**b**) Tunneling spectra along the zigzag edge at locations A, B, C, D, E, and F of Fig. 3a. The tunneling gap was stabilized at *V*_*s*_ = 1.0 V, and *I*_*t*_ = 0.5 nA before spectra were taken under constant height mode. These spectra overlap nicely, showing reproducibility. (**c**) Tunneling spectra measured at different positions between the zigzag edge and the bulk obtained at locations 1, 2, 3, 4, 5, and 6 in Fig. 3a. (**d**) Difference spectra, *dI/dV* subtracted from the bulk *dI/dV*, obtained at locations 1, 2, 3, 4, 5, and 6 in Fig. 3a.The spectrum at location 6 is used as the bulk reference.

**Figure 4:**
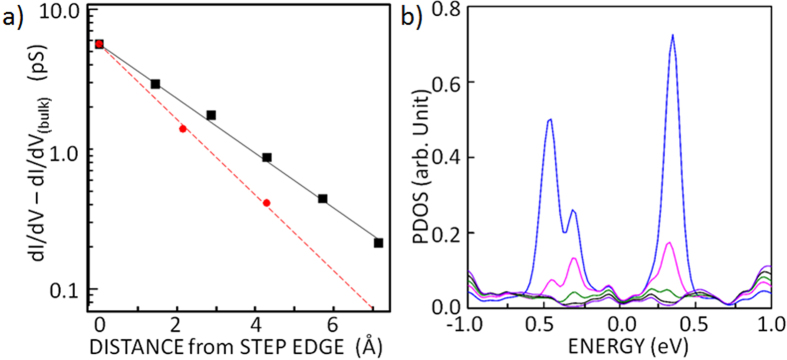
Measured decaying DOS from a zigzag edge to the bulk of a graphene layer. (**a**) Measured (full black squares) and calculated (full red circles) difference in *dI/dV* (decaying edge state) at locations away from a zigzag edge. (**b**) Theoretical *k* dependence of partial DOS with on-site Coulomb repulsion at the zigzag edge (blue) and at 3 lattice constants (red), 6 lattice constants (green), 9 lattice constants (black), and 12 lattice constants (purple) from the edge.

Three different electronic structures are expected in a graphene layer on *h*-BN, as illustrated in Fig. 2a: a bulk graphene spectrum with a possible bulk bandgap, zigzag gap states with a different bandgap, and an armchair edge-resonance spectrum with the same bandgap as that of the bulk. Figure 2b,c show STM images acquired near the observed zigzag and armchair edges, respectively. Atomic details are visible with moiré patterns as their minima are indicated by blue arrows. The lengths of the zigzag edges range from 10 nm to 100 nm without a single defect or reconstruction. At the armchair edges on an *h*-BN layer, many defects are present, unlike for free-standing or epitaxial graphene layers on a metal surface. Considering this observation, the zigzag edges seem to be energetically more stable than armchair edges in the graphene layer on *h*-BN. The spectra change to the bulk spectra with a short decay length near the zigzag edges (0.28 nm, to be discussed in Figs 3 and 4) and with a slowly decaying beat pattern near the armchair edges (5–10 nm)^31^,^32^. Figure 2d shows a spectrum acquired at a distance of five lattice constants from a zigzag edge (full green circles) in comparison with a spectrum taken at 2.4 nm from the edge (full dark-red circles). The two spectra are almost identical to the bulk spectrum in Fig. 1b and deviate from the Dirac dispersion; two peaks at ≈−0.14 eV and ≈−0.21 eV from the long-range moiré potential, and two bumps protruded from an imaginary straight Dirac dispersion. Only after subtracting a straight Dirac dispersion and a parabolic tunneling I-V background in a tip-sample geometry from the spectra, two features centered at (−0.05 ± 0.02) eV and (0.05 ± 0.02) eV are visible as shown in the inset of Fig. 2d. This observation hints an evidence of a bulk gap-like feature. The gap is far less (≤1/2) than the separation between these two features when the instrumental broadening of peaks and the background are considered. The measured gap in a recent transport measurement^5^ was 10–30 meV with various twist angles between the *h*-BN and the graphene layers. A gap of 10–50 meV was predicted by theory^4^,^6^,^7^.

### Armchair Edge Resonance

Near the armchair edges, charge density (height in a constant current STM image) modulates within a period of 10–16 lattice constants (Figs 2c and S6) because the intervalley scattering is only allowed at an armchair edge^31^,^32^. In STS results, two strong peaks appear at −0.07 ± 0.01 eV and at 0.04 ± 0.01 eV^31^ at an armchair edge (full blue circles in the STM image in Fig. 2c and spectra Fig. 2d) and at the charge-density peak near that armchair edge (full black circles in the STM image in Fig. 2c and spectra in Fig. 2d), respectively, and another charge-density peak appears near another armchair edge (not shown in Fig. 2c and full red circles in spectra in Fig. 2d). The positions and strength of these armchair edge resonances varied with armchair edge shape and roughness. The origin of the two strong peaks could only be explained theoretically in a GNR (Fig. S7), but not straightforwardly in GNIs. A perfect armchair graphene edge may reveal the bulk bandgap but no localized edge state since its one-dimensional bulk Hamiltonian is topologically trivial for any k-perpendicular value. But reconstructed armchair edges may have localized edge states due to the modified geometries and the resultant hopping properties. This result was demonstrated with the complex band structure of graphene in the bulk^33^. This scenario may explain the spectra with small edge roughness or edge relaxation. Several extra peaks were observed only in the spectrum at the armchair edges (full blue circles in Fig. 2d) in addition to the two peaks. However, these extra peaks decay within 0.5 nm, implicating edge defects or relaxation as a cause of the extra peaks.

### Zigzag Edge States

Figure 3a shows a close-up view of a zigzag edge. The curved feature at the edge is due to the finite curvature (≈1 nm) of the tunneling tip used to obtain this STM image. The inset in Fig. 3a is a simulated STM image of graphene on *h*-BN using a tight-binding calculation; the simulated image is very similar to the observed STM image. In a GNR with zigzag edge terminations, the tight-binding calculation predicted a flat band at the Fermi level between 2/3*k*_Γ*X*_ and *k*_Γ*X*_ in momentum space where *k*_Γ*X*_ = 3/4*k*_Γ*X*_ and *k*_Γ*X*_ is the crystal momentum in the direction from Γ to X in the first Brillouin zone. Unlike GNR, in the case of a GNI with a long zigzag edge, the states at the Dirac point are expected to be reduced but still present^11^. At a zigzag edge, an energy gap may open to reduce the Fermi instability when considering the spin degree of freedom. The calculated bandgap varies as a function of the crystal momentum. Meanwhile, if there is no corresponding state in the bulk, this gap state decays from the zigzag edge toward the bulk. Figure 3b shows the measured tunneling spectra at six equivalent carbon sites along the edge (at sites A,B,C,D,E,F). The spectra overlap nicely with peaks at −0.3–−0.15 eV. The origin of these peaks can be explained with the long-range moiré potential, but the details are not explained. Figures 3c and S8 show the tunneling spectra measured at six sites (1,2,3,4,5) between the edge and the bulk at 1.42 Å intervals. These spectra also overlap nicely, with the exception of the energy between −0.1 eV and +0.2 eV. The measured density of states is higher at the edge than in the bulk near the Fermi level (*dI/dV* at the Fermi level is higher at the edge than in the bulk near the Fermi level), suggesting presence of the edge state. Therefore, the difference between the edge and the bulk is more meaningful than small features at ≈0.05 eV, ≈0.10 eV and ≈0.15 eV at the edge (location 1). Figure 3d shows the first derivative of the tunneling current (*dI/dV*) subtracted from the bulk *dI/dV*. These difference spectra clearly show the presence of the edge state at this zigzag edge. The measured edge state at the Fermi level decreases with increasing distance from the edge. The height, and therefore, the strength of the edge state decreases with increasing distance from the zigzag edge. A gap-like feature is visible near the Fermi level, and the size of this gap does not vary with distance from the edge of the bulk stripe. The peak-to-peak energy gap was estimated to be ≈(0.09 ± 0.01) eV, as shown in Fig. 3d.

## Discussion

The peak of the difference spectra, *dI/dV* subtracted from the bulk *dI/dV*, is plotted in Fig. 4a as a function of the distance from the edge. The decay of the edge state is nearly exponential with a decay length of ≈2.8 Å (full black squares) in the semi-log plot of difference in *dI/dV* versus distance. An *ab initio* calculation was performed to calculate the edge state at five equal distances from the zigzag edge with a periodic boundary of 4 nm (Fig. 4b). The calculated results show similar decay behavior (Fig. S9). By plotting the calculated results (full red circles) in the same semi-log plot in Fig. 4a, the decay length was found to be overestimated by ≈1 Å in the calculated result. This discrepancy may be caused by the fact that the GNI was modeled by a GNR with a width of 40 Å. The zigzag edge states decay toward the bulk because they do not have corresponding states in the bulk. The decay length of the zigzag edge state can be estimated using the complex band structure^33^, although the real part of the wave number is only considered in a bulk crystal under the Born–von Karman boundary condition. The imaginary part of the wave number corresponds to the inverse of the decay length. The calculated decay length is ≈2 Å, which is in a good agreement with the measured one.

**Figure 5:**
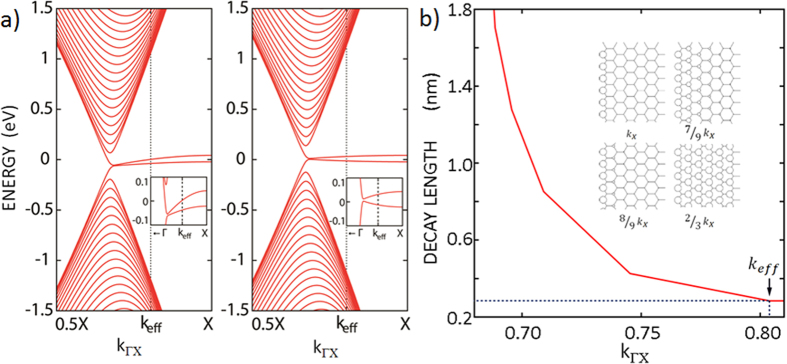
Theoretical *k* dependence of the decay length and calculated band structure with on-site Coulomb repulsion in the tight-binding model. (**a**) Band structures calculated using the tight-binding model with on-site Coulomb repulsion terms only at the zigzag edges, showing metallic edge states. When the calculation was performed with on-site Coulomb repulsion terms at all carbon sites, a semiconducting edge state was obtained. (**b**) Momentum-dependent decay length based on the tight-binding calculation. Compared with the experimental value, the effective *k* value of the current tip is 0.81*k*_*ΓX*_. The inset shows the decay length dependence of the *k* value^17^. The open circles represent edge states.

We calculated two band structures considering the on-site Coulomb repulsion terms only at the edge, showing a metallic character (*k* = *k*_Γ*X*_); left side of Fig. 5a) and at all carbon sites (*k* = 2/3*k*_Γ*X*_), showing a semiconducting character (right side of Fig. 5a). On the basis of this result, one must consider the on-site Coulomb repulsion terms in most of the carbon sites to explain the decay result in Figs 3d and 4a. By varying the |*U/t*| ratio in the Hubbard-model Hamiltonian, the |*U/t*| value of 0.1 explained our data well as described in the supplementary information.

In the STM and STS measurements, quantum tunneling has a heavier weight factor along the Γ or the Brillouin zone boundary direction than it does in other k directions^34^. However, in some tunneling tips, electrons may prefer to tunnel to states at k between Γ to the Brillouin zone boundary. In these cases, the measured decay length may vary with different tip electronic structures in addition to the edges state effect in Fig. 4ab. We can model the dependence in a GNR with two zigzag terminations. Again, the GNI can be modeled as a GNR when the length of the edge is large^11^. In the tight-binding calculation shown in the inset of Fig. 5b^17^, the edge state is present in all the carbon sites at the momentum *k* = 2/3*k*_Γ*X*_, and the gap state is localized only at the zigzag edge at the momentum *k* = *k*_Γ*X*_. This dependence is shown as a red solid-line in Fig. 5b. The measured decay length of 2.8 Å is plotted as a black dotted-line in Fig. 5b. By comparing the theoretical and measured decay lengths, we were able to extract the effective *k* value of the measuring probe tip, 0.81*k*_Γ*X*_, in consideration of the tip artifact while ignoring the edge state effect. We found that the decay length varies for different tips, thus confirming the tip effect.

The spin ground state of a zigzag edge can be imaged using a spin-polarized STM. We attempted this measurement on several graphene islands grown on the *h*-BN layer. Despite reproducible measurements of the zigzag gap states, spin-polarized signals were not detected. As suggested by theoretical predictions^14^, the antiferromagnetic state may be the ground state in a GNR; however, this phenomenon could not be detected in the present experimental geometry because most graphene islands were irregularly shaped, non-parallel nanoribbons. We found edge relaxations as large as ≈4%. Exact measurements of the energy gap at the zigzag edge may lead to a new perspective for understanding the transport results in the GNR, as suggested by recent experiments^35^.

## Methods

The graphene on the *h*-BN films was epitaxially grown in an ultrahigh vacuum chamber connected to a homemade low-temperature scanning tunneling microscope. Prior to deposition, a Cu(111) single crystal was cleaned by repeated *Ar*^+^ sputtering and annealing cycles. Sub-monolayer *h*-BN was grown using borazine (B_3_H_6_N_3_) at 10^‒5 ^Pa while the sample was maintained at a temperature of 1000 K. Ethylene (C_2_H_4_) at 10^‒3^ Pa was introduced to form graphene on the half-monolayer *h*-BN-covered Cu(111) surface at 1100 K. The sample was maintained at that temperature for 2 h and then cooled at a rate of 30 K/s. All STM and STS measurements were conducted at approximately 4.9 K. STM imaging was performed under constant current mode, whereas STS was performed under constant height mode.

Density functional theory (DFT) calculations were performed within the generalized gradient approximation (GGA) using the Vienna ab initio simulation package (VASP). The projector-augmented wave potentials, as implemented in VASP, were employed to describe DOS at atom centers. The energy cutoff for the plane-wave basis set was adjusted to 400 eV in the GGA. The model structures were optimized until the Hellman–Feynman forces acting on the atoms became lesser than 0.01 eV/Å. To include the weak van der Waals interactions between adsorbates and graphene, we adopted Grimme’s DFT-D2 correction based on a semi-empirical GGA-type theory. For Brillouin zone sampling, we used a 27 × 27 × 1 grid in the Monkhorst-Pack special k-point scheme. In addition to the DFT calculation, we also performed a tight-binding calculation with the Hubbard−model Hamiltonian for zigzag GNRs with various widths (3.4–34 nm). The ratio of the on-site repulsive potential U to the hopping parameter *t* in the model Hamiltonian was fitted using the measured energy gap and a hopping parameter of *t* = −2.88 eV. The effective wavevector *k* adapted to the observed decay length of the edge state was obtained from the analytical solution for the states localized at the zigzag edge. Certain commercial equipment, instruments, or materials are identified in this paper in order to specify the experimental and theoretical procedures adequately. Such identification is not intended to imply recommendation or endorsement by the National Institute of Standards and Technology, nor is it intended to imply that the materials or equipment identified are necessarily the best available for the purpose.

## Additional Information

**How to cite this article**: Hwang, B. *et al*. Energy Bandgap and Edge States in an Epitaxially Grown Graphene/*h*-BN Heterostructure. *Sci. Rep.* **6**, 31160; doi: 10.1038/srep31160 (2016).

## Supplementary Material

Supplementary Information
