# Supplementary material for: Energy Bandgap and Edge States in an Epitaxially Grown Graphene/h-BN Heterostructure
Source: Sci Rep. 2016 Aug 9;6:31160. doi: 10.1038/srep31160 (PMC4977565; doi:10.1038/srep31160)
Supplement: Supplementary Information [file srep31160-s1.pdf]

Supplementary Information

Energy Bandgap and Edge States in an Epitaxially Grown Graphene/h-BN Heterostructure

Beomyong Hwang*, Jeongwoon Hwang*, Jong Keon Yoon*, Sungjun Lim*,Sungmin Kim*, Minjun Lee*, Jeong Hoon Kwon*, Hongwoo Baek*, , Dongchul Sung+, Gunn Kim+, Suklyun Hong+, Jisoon Ihm*, Joseph . A. Stroscio‡ and Young Kuk*

**Department of Physics, Seoul National University, Seoul 151-747, Korea, +Department of Physics, Sejong University, Seoul 143-747, Korea, ‡Center for Nano Science and Technology, National Institute of Standards Technology, Gaithersburg 20899, MD*

S1. Growth of Graphene Layer on grown *h*-BN on Cu(111)

A graphene layer can be grown on a hexagonal boron nitride (*h*-BN) substrate by introducing precursor gases (ethylene) at 10^8–10^9 Langmuir (L)[1-4]. To produce a clean graphene surface and interface with *h*-BN, we grew graphene monolayers on an *h*-BN monolayer with ethylene as the precursor gas at 10^3–10^4 L. Meanwhile we could lower the growth temperature by ionizing the precursor gas by turning on a plasma source. Figures S1-S2 show grown graphene islands on a partly covered *h*-BN layer with the ethylene dose of ≈10^3 L. The substrate Cu surface is partly covered on an *h*-BN monolayer to expose the bare Cu surface. The precursor gas molecules can dissociate on the bare Cu surface, and the carbon atoms diffuse along the step edges of *h*-BN. The chemical identification of the graphene, Cu and *h*-BN surfaces was confirmed by the scanning tunneling spectra, as shown in Fig. 1b. More than a dozen different

moiré patterns were observed in *h*-BN layers; however, some *h*-BN layers did not reveal any moiré pattern. As the growth rate of the graphene layers was extremely low on the *h*-BN surface at the present exposure, grown graphene islands were scarce. Owing to the electric structure of graphene and *h*-BN, the moiré patterns of *h*-BN layers were imaged at the tunneling bias voltage of ~4 V.

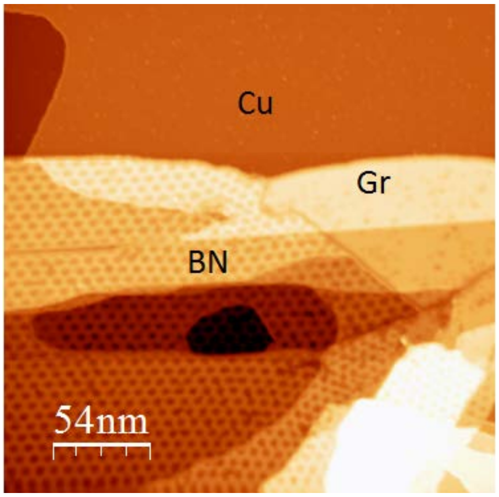

Fig. S1: STM topography of a (260 × 260) nm^2 *h*-BN covered surface obtained with V_s = 4.0 V,

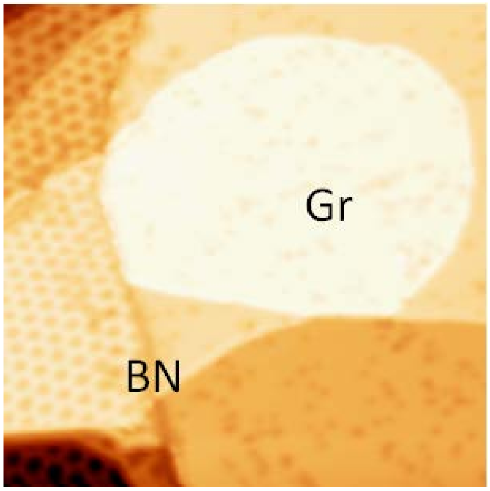

Fig. S2: STM topography of a (130 × 130) nm^2 *h*-BN covered surface obtained with V_s = 4.0 V.

S2. Decaying charge densities and simulated STM images

The electronic properties of graphene directly grown on a metallic substrate are quite different from those on an insulator surface. To investigate the influence of the metallic substrate, we calculated the charge densities toward the surface normal, as shown in a log scale for the two peaks near the Fermi energy in Figs. S3a, and S3b. To see the spatial variation on the graphene surface, we calculated the charge density perpendicular to a carbon (marked C) and a honeycomb hollow (marked h) sites. In the case of the graphene/Cu(111) system, there are two dominant states originated from the Cu s-orbital and C p-orbital. The electronic states in the range of ±25 meV from the peaks were integrated in the calculation of the charge densities. Without an insulating *h*-BN layer between graphene and Cu, the Cu s-orbital dominant state decays very slowly showing its tail above the graphene layer as shown S3a. By inserting an *h*-BN layer between graphene and Cu, the contribution from Cu s-orbital is reduced and that from C p-orbital dominate the measured charge as shown in Fig. S3b.

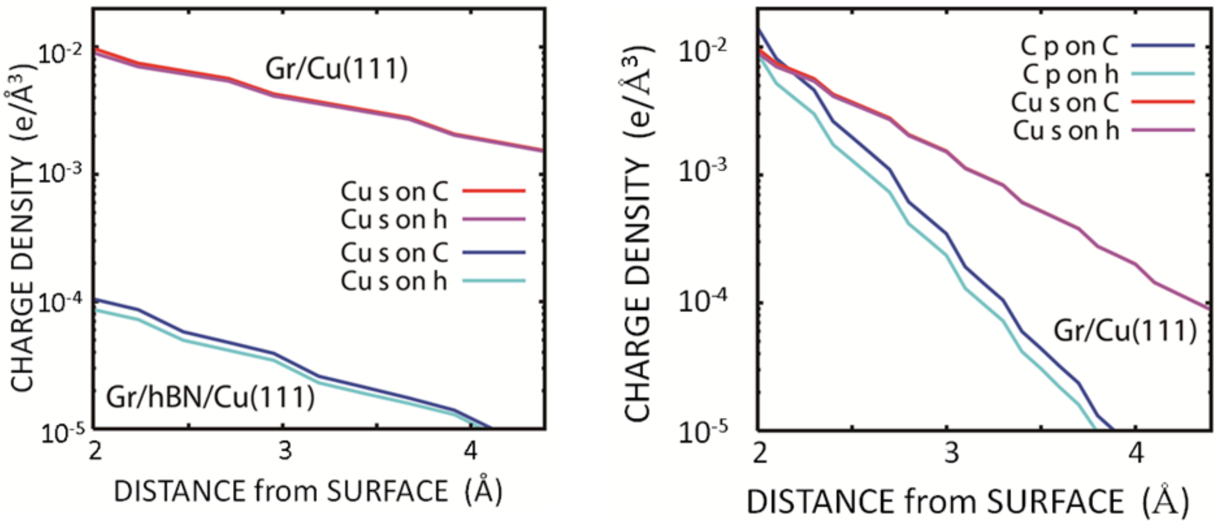

Fig. S3: Calculated charge density away from the graphene surface. a) Cu s states from carbon sites and hollow sites on graphene on Cu(111) and graphene on *h*-BN on Cu(111). b) Cu s states and carbon p states from on graphene directly grown on Cu(111)

To simulate the spatial distribution of the measured charge density, we integrated the charge densities from −0.05 to 0.00 eV on pristine graphene (Figs. S4a and S4b), and on graphene/*h*-BN/Cu(111) (Figs. S4c and S4d). Considering an STM lateral resolution of 1 Å, the charge densities were averaged within a circle with a radius of 1 Å at a given height. The locations of the Cu substrate, B and N are marked. In the case of pristine graphene, the bonding sites are brighter in the energy range while one of the two sub-lattices is brighter on the *h*-BN/Cu(111) because of the sub-lattice effect.

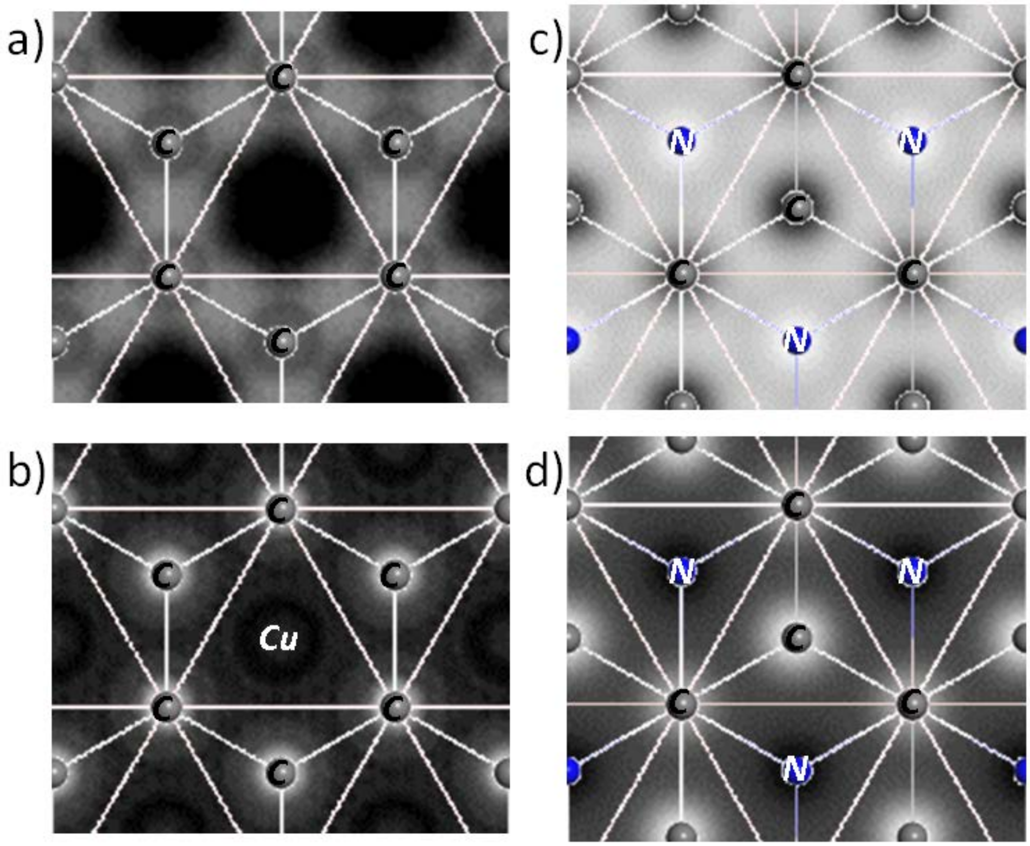

Fig. S4: Spatial distribution of the charge densities at the heights of 3 Å (a) and 4 Å (b) away from the graphene layer grown on Cu(111) and the heights of 3 Å (c) and 4 Å (d) away from the graphene layer grown on *h*-BN layer on Cu(111).

S3. Calculation of the band structure

The density functional theory (DFT) calculation was performed within the generalized gradient approximation (GGA) using the Vienna ab initio simulation package (VASP)[33]. The projector−augmented wave potentials, as implemented in the VASP, were employed to describe with respect to the atom centers. The calculated graphene band gap was 0.11 eV.

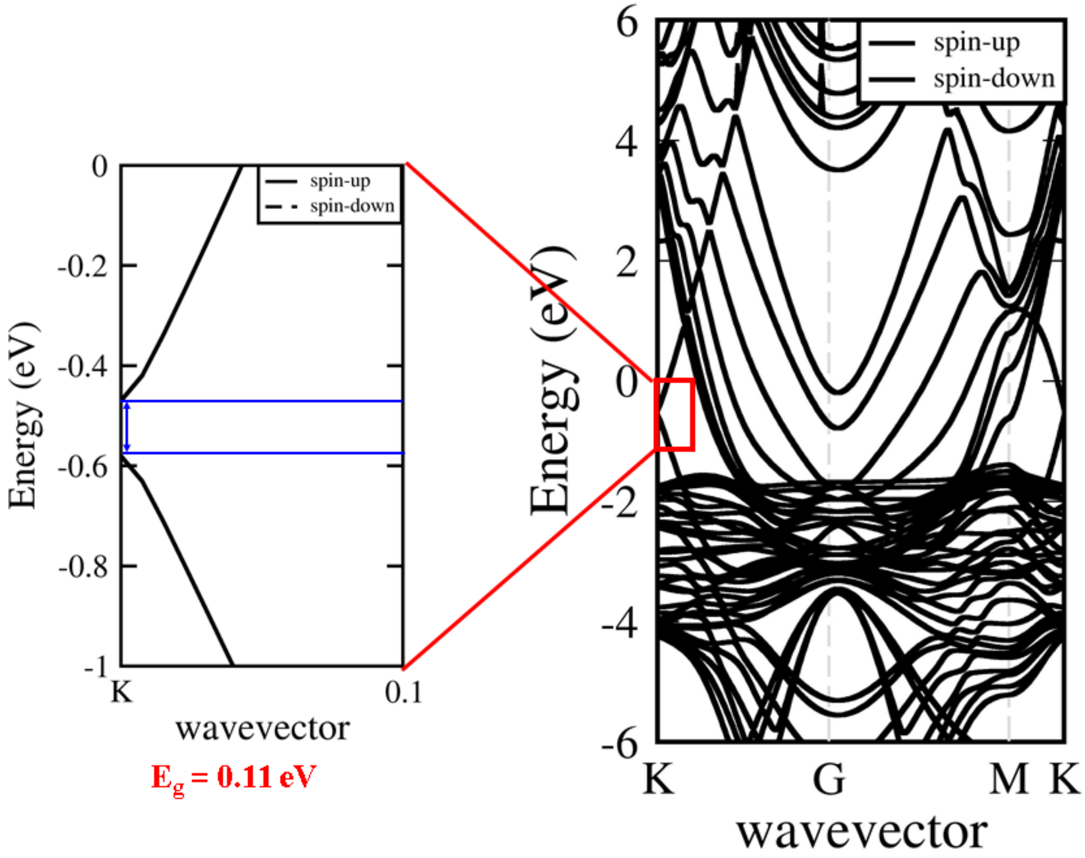

Fig. S5: Graphene/ *h*-BN/ Cu(111) band Structure

S4. Charge density interference near armchair edges

Near armchair edges, charge density (height in a constant current STM image) modulates with a period of 10 to 16 lattice constants as shown in Fig. 2c and in Fig. S5 because the intervalley scattering is only allowed at an armchair edge[29,30]. Figures S5a and S5b show near-sinusoidal intensity variation near armchair edges. These observations are originated from intravalley scattering prohibited at armchair edges.

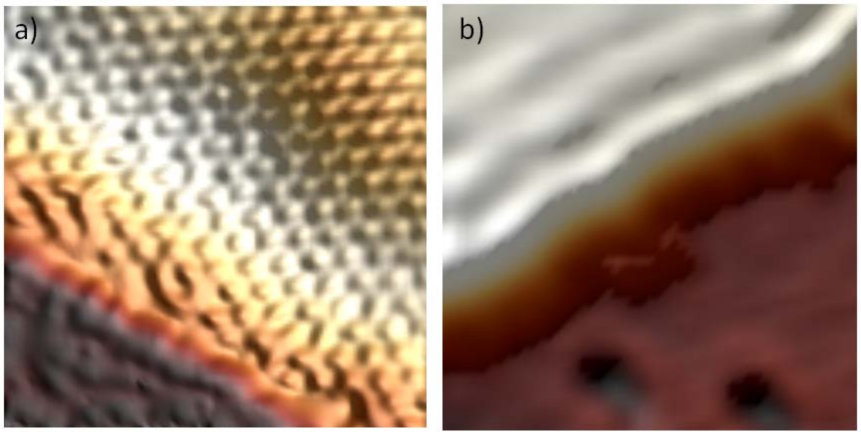

Fig. S6. Topography images at two different armchair edges. a) 3.5 × 3.5 nm^2 image at V_s = 20 mV and I_t = 20 pA. b) 6.5 × 6.5 nm^2 image at V_s = 100 mV and I_t =0.1 nA.

S5. Electronic structure near an armchair edge

The density functional theory (DFT) calculation was performed within the generalized gradient approximation (GGA) using the Vienna ab initio simulation package (VASP)[33] to compare with the measured spectra near an armchair edge. Calculated results were obtained as carbon atoms in the graphene layer were placed on B atoms and hollow sites in *h*-BN layer. Calculated charge densities at five locations from the edge are shown with five different colors in

the atomic structures and spectra. Two strong peaks are reproduced. However, the peak width decreases with the size of the armchair nano ribbon. We expect that the gap would converge with wide armchair.

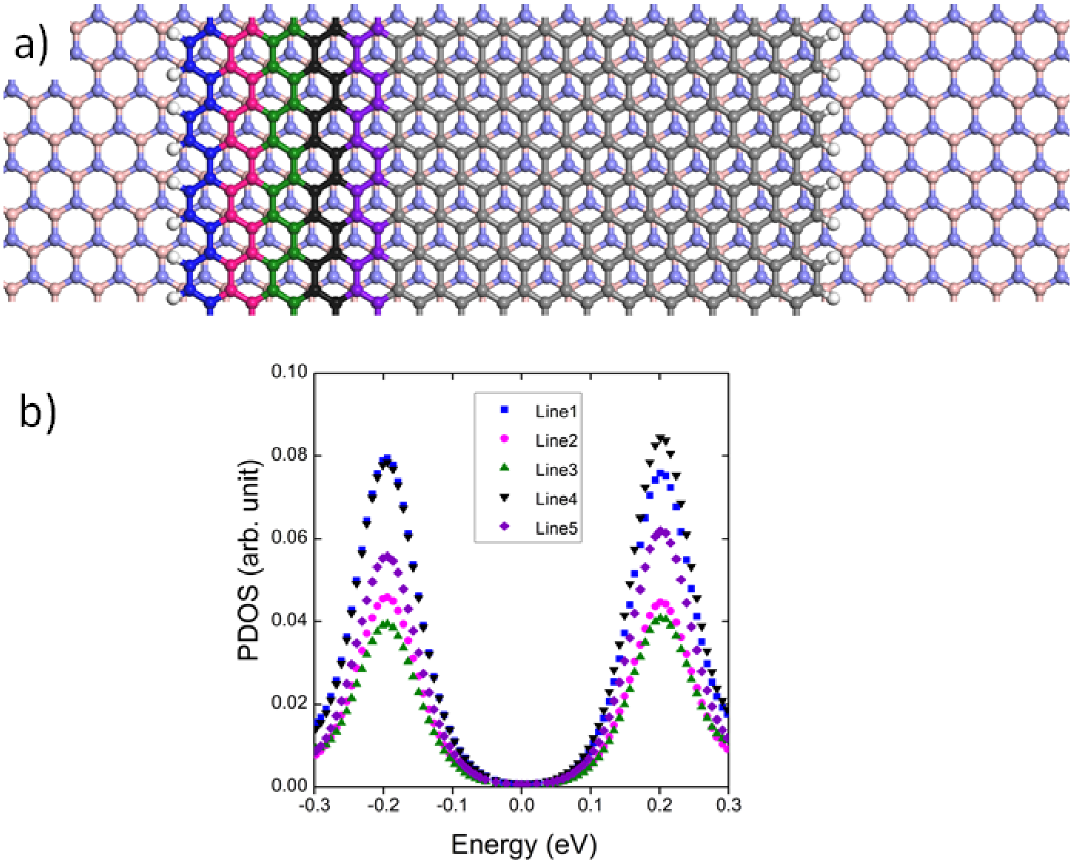

Fig. S7. Armchair terminated nano ribbon on the *h*-BN layer with a width of 3.6 nm. A Gaussian broadening sigma of 0.03 was used to show the calculated spectra.

S6. Positional dependence of tunneling spectroscopy data near a zigzag edge

Figure S6 shows the spatial variation of the tunneling spectroscopy data near a zigzag edge. The reproducibility is as good as those in Figs. 3b and 3c. Unlike Fig. 3b, the spectra were taken

only at carbon sites. The spectra at carbon sites show similar structures; However, the decay of edge states between −0.1 eV and +0.2 eV is clearly visible as shown in Figs. 3b and 3c. The decay behavior is exactly the same at carbon sites and hollow sites of the honeycomb lattice. The measured density of states (DOS) is higher at the edge than at the bulk state at the Fermi level. The measured edge state at the Fermi level decreases with increasing distance from the edge.

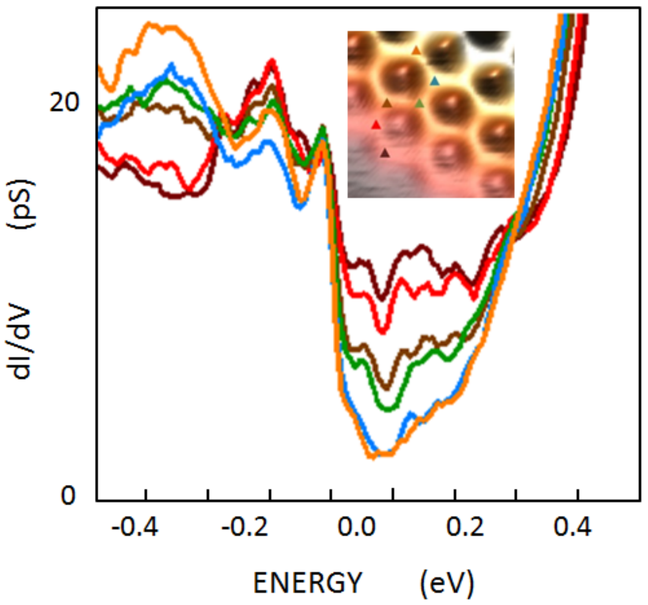

Fig. S8: Scanning tunneling spectroscopy data near a zigzag edge.

S7. Decaying zigzag states

The decay length of the zigzag edge state can be estimated by using the complex band structure[32], although real part of wave numbers is only considered in a bulk crystal under the Born–von Karman boundary condition. The imaginary part of the wave number corresponds to the inverse of the decay length. The calculated decay length is ≈2 Å, showing a good agreement with measured one. Decaying zigzag states are calculated with three different Gaussian

broadening parameters. The peak height varies slightly, but the summarized data in Fig. 4a do not change. Experimental data agree well with Gaussian broadening sigma of 0.03.

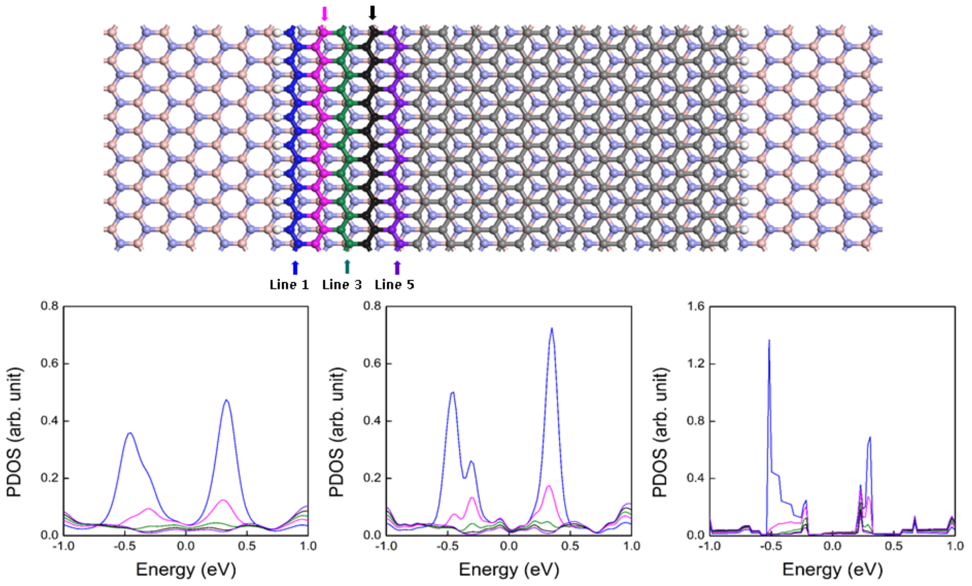

Fig. S9. Decaying edge states at the zigzag edge with three different Gaussian broadening sigma equal to 0.05, 0.03 and 0.00 from left to right.

Supplementary References

1. M. Wang et al. “A Platform for Large-Scale Graphene Electronics – CVD Growth of Single-Layer Graphene on CVD-Grown Hexagonal Boron Nitride,” Adv. Mater. **25**, 2746-2752 (2013)

2. S. Loth et al. “Chemical Vapor Deposition and Characterization of Aligned and Incommensurate Graphene/Hexagonal Boron Nitride Heterostack on Cu(111),” Nano Lett. **13**, 2668-2675 (2013)

3. W. Yang et al. “Epitaxial growth of single-domain graphene on hexagonal boron nitride,” Nature Mater. **12**, 792-797 (2013)

4. G.H. Han et al. “Continuous Growth of Hexagonal Graphene and Boron Nitride In-Plane Heterostructures by Atmospheric Pressure Chemical Vapor Deposition,” ACS Nano **7**, 10129-10138 (2013)
